# Arthritis of the sternoclavicular joint masquerading as rupture of the cervical oesophagus: a case report

**DOI:** 10.1186/1752-1947-3-40

**Published:** 2009-01-29

**Authors:** Iraklis E Katsoulis, Manuela Bossi, Nisal Damani, Jeremy I Livingstone

**Affiliations:** 1 Upper Gastrointestinal Surgery Unit, Watford General Hospital, Watford, Hertfordshire, UK; 2 Department of Radiology, Watford General Hospital, Watford, Hertfordshire, UK

## Abstract

**Introduction:**

Sternoclavicular septic arthritis is a rare condition and accounts only for 1% of cases of septic arthritis in the general population. The most common risk factors are intravenous drug use, central-line infection, distant-site infection, immunosuppression, trauma and diabetes mellitus. This is a report of an unusual case where this type of arthritis was masquerading as rupture of the cervical oesophagus.

**Case presentation:**

A 63-year-old man presented complaining of right neck pain and dysphagia following a bout of violent coughing. Physical examination revealed cellulitis extending from the right sternoclidomastoid region to the anterior upper chest. Computed tomography showed inflammatory changes behind the right sternoclavicular joint with mediastinitis and ipsilateral pleural effusion. These findings raised the suspicion of spontaneous rupture of the cervical oesophagus. Management involved jejunal feeding along with broad-spectrum antibiotics. The inflammation, however, relapsed after discontinuation of the antibiotics and this time, computed tomography pointed to a diagnosis of arthritis of the sternoclavicular joint. The patient responded completely to a 6-week course of oral penicillin, flucloxacillin and metronidazole.

**Conclusion:**

Sternoclavicular arthritis is a rare condition that has been associated with a variety of predisposing factors. It may, however, occur in otherwise completely healthy individuals and should be included in the differential diagnosis of other inflammatory conditions of the neck and upper chest.

## Introduction

Sternoclavicular septic arthritis is a rare condition and accounts only for 1% of cases of septic arthritis in the general population [1]. The most common risk factors are intravenous drug use, central-line infection, distant-site infection, immunosuppression, trauma and diabetes mellitus. It has, however, been described in previously and otherwise healthy adults without associated predisposing risk factors [2].

Due to the anatomical location of the infection, this entity can often mimic other inflammatory conditions of the neck and upper chest. This is a report of sternoclavicular arthritis in a previously healthy adult that was initially misdiagnosed and managed as possible spontaneous rupture of the cervical oesophagus.

## Case presentation

**Figure 1:**
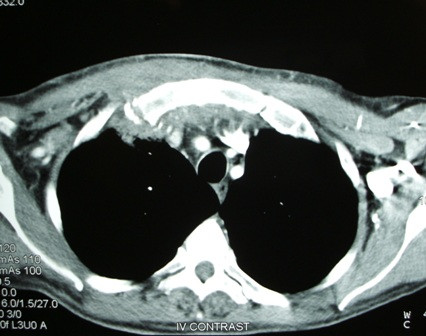
**Computed tomography scan showing inflammatory changes behind the right sternoclavicular joint with small pockets of air behind the upper sternum**.

**Figure 2:**
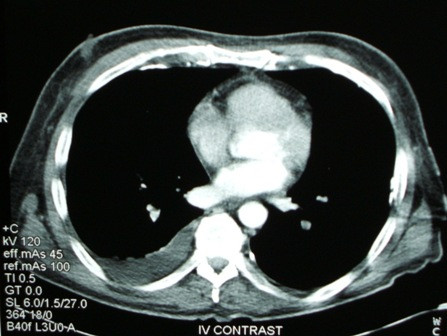
**Computed tomography scan showing right pleural effusion**.

A 63-year-old Caucasian man presented complaining of right neck pain and dysphagia following a bout of violent coughing that he experienced on the previous day. On admission, he was haemodynamically normal with mild pyrexia. Physical examination revealed cellulitis extending from the right sternoclidomastoid region to the anterior upper chest with swelling and tenderness just above the right sternoclavicular joint. The blood tests showed leukocytosis and raised inflammatory markers. A plain chest X-ray showed a right pleural effusion. These findings raised suspicion of a spontaneous rupture of the cervical oesophagus. Oral intake was omitted and a nasojejunal tube was inserted for feeding along with empirical intravenous administration of penicillin, flucloxacillin and metronidazole. A computed tomography (CT) scan showed inflammatory changes behind the right sternoclavicular joint with small pockets of air behind the upper sternum, pleural thickening at the right apex with some adjacent lung consolidation and confirmed the presence of a pleural effusion (Figures 1 and 2).

**Figure 3:**
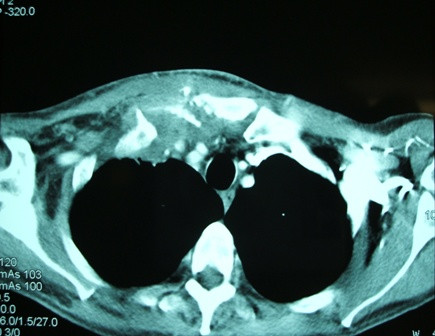
**The final computed tomography scan established the correct diagnosis, showing erosive changes within the joint**.

A gastrografin swallow study showed a small irregularity of the lateral pharyngeal wall but not definitive contrast leak, and pharyngoscopy could not find any abnormality. Paracentesis of the sternoclavicular swelling was attempted under ultrasound guidance, but no micro-organisms were isolated from the aspirate. Nevertheless, because there was still a degree of uncertainty, it was decided to treat the condition with prolonged jejunal feeding and antibiotics. After 2 weeks on this regime, the inflammation resolved completely and the patient was allowed oral feeding and discharged home. However, 9 days after his discharge, the patient presented with the same symptoms. Another CT scan established the diagnosis of septic arthritis showing erosive changes within the right sternoclavicular joint (Figure 3). The patient restarted a 6-week course of the same antibiotic combination but without restrictions in oral intake. Eventually, the arthritis settled and on follow-up 3 and 6 months later, respectively, the patient remained asymptomatic.

## Discussion

Infection of the sternoclavicular joint is an uncommon entity often misdiagnosed at the first presentation. In 2004, Ross et al. published a comprehensive review of 180 cases [1]. Sternoclavicular arthritis accounted for 1% of all cases of septic arthritis. Common risk factors included intravenous drug use (21%), distant site of infection (15%), diabetes mellitus (13%), trauma (12%) and infected central venous line (9%). No risk factor was found in 23% of cases.

Bacteria can easily pass from the subclavian vein into the joint space. This explains how a central-line insertion or drug injection directly into the jugular vein can cause the infection. For the same reason, a sternoclavicular infection can be secondary to a skin infection. Furthermore, various distant sites of infection have been described: urinary and respiratory tract infections, pericarditis and intra-abdominal abscess. *Staphylococcus aureus *is responsible for the majority of the cases (49%), followed by *Pseudomonas aeruginosa *(10%), *Brucella melitensis *(9%), *Escherichia coli *(5%) and *Mycobacterium tuberculosis *(3%). Most cases of *Pseudomonas *pyarthrosis affecting the sternoclavicular joint have been reported in immunosuppressed intravenous drug users [3]. However, due to paucity of joint fluid, sometimes the culture from the needle aspiration may be negative [1]. Serious complications such as osteomyelitis (55%), chest wall abscess or phlegmon (25%) and mediastinitis (13%) are common. Another potentially life-threatening complication is the formation of a retrosternal abscess [4].

The classical clinical presentation of patients with septic sternoclavicular arthitis involves an insidious onset of chest pain localized to the sternoclavicular joint or pain referred to the ipsilateral shoulder or neck. Fever and leukocytosis are not invariably present. The median duration of symptoms at presentation is much longer than the typical septic arthritis of other joints. The majority of patients with this condition have a normal plain radiograph. The plain chest X-ray in our case showed contra-lateral deviation of the trachea and an ipsilateral pleural effusion. A CT scan showed inflammatory changes behind the right sternoclavicular joint, pockets of air behind the upper sternum, pleural thickening at the right apex with some adjacent lung consolidation. It also confirmed the tracheal deviation and the pleural effusion. CT scans show osteomyelitis in 69% of the reported cases, chest-wall abscess or phlegmon in 57%, joint space widening or fluid in 25% and mediastinitis in 20% [5]. The association of pleural effusion with sternoclavicular arthritis has only been reported once before in the medical literature [6].

Our case raised the suspicion of oesophageal rupture mainly due to the history of violent cough and the anatomical distribution of the inflammation associated with mediastinitis and pleural effusion. In fact, forceful increase of the pharyngeal pressure may cause spontaneous rupture of the cervical oesophagus [7]. The atypical clinical and radiological presentation led to a wrong diagnosis, and the management involving restricted oral intake and jejunal feeding proved unnecessary. Eventually, the correct diagnosis was made and the patient responded completely to treatment with long-term oral antibiotics.

Prompt diagnosis and appropriate treatment with long-term antibiotics result in an excellent outcome in most cases. The choice of the broad-spectrum combination penicillin, flucloxacillin and metronidazole in our case was empirical, given that no risk factors for this condition were identified and no micro-organisms were isolated in the joint aspirate. Sternoclavicular septic arthritis may occasionally require operative treatment with resection of one half of the manubrium and the medial third of the clavicle. This may be indicated either when the arthritis involves extensive bony destruction or when conservative management is unsuccessful [1]. Operative drainage and debridement are also mandatory in cases of retrosternal abscess formation [4].

## Conclusion

Although rare, sternoclavicular arthritis can mimic other inflammatory conditions of the neck and upper chest due to the anatomical location of the infection and may be misdiagnosed, especially in patients without associated risk factors.

## Abbreviations

CT: computed tomography.

## Consent

Written informed consent was obtained from the patient for publication of this case report and accompanying images. A copy of the written consent is available for review by the Editor-in-Chief of this journal.

## Competing interests

The authors declare that they have no competing interests.

## Authors' contributions

IEK conceived the idea, collected the patient's data and was the major contributor to the writing of the manuscript. MB did the literature search and drafted the manuscript. ND performed the CT examinations and JIL critically reviewed the paper. All authors read and approved the final manuscript.
